# Role of netrin-1 and netrin-1 dependence receptors in colorectal cancers

**DOI:** 10.1038/sj.bjc.6602656

**Published:** 2005-06-14

**Authors:** P Mehlen, F Llambi

**Affiliations:** 1 Apoptosis, Cancer and Development Laboratory, Laboratoire labellisé ‘La Ligue’, CNRS FRE2870, Centre Léon Bérard, 28 rue Laennec, 69008 Lyon, France

**Keywords:** dependence receptor, apoptosis, colorectal cancer

## Abstract

Although cancer is a multifaceted disease, all cancer types share identical molecular and cellular mechanisms. These mechanisms involve a collection of alterations critical to the normal physiological functioning of cells, such as alterations of growth factor signalling pathways, angiogenesis, cell adhesion signals, DNA replication and apoptotic cell death. Many genes involved in the processes enumerated above are functionally inactive in tumour cells, designating them as putative ‘tumour suppressor genes’. Back in the early 1990s, Vogelstein and colleagues suggested that a gene called DCC (for Deleted in Colorectal Cancer) could be a tumour suppressor gene because it was found to be deleted in more than 70% of colorectal cancers, as well as in many other cancers. During the last 15 years, controversial data have failed to firmly establish whether DCC is indeed a tumour suppressor gene. However, the recent observations that DCC triggers cell death and is a receptor for netrin-1, a molecule recently implicated in colorectal tumorigenesis, have prompted a renewal of interest in the role of DCC in tumorigenesis and suggest that the netrin-1/receptor pairs act as novel negative regulators of tumour development.

While the classic view of receptor function is mono-sided, that is, a receptor is inactive until bound and activated by its ligand, during the last decade it has been suggested that some receptors may display two sides, à la ‘Jeckyll and Hyde’: in the presence of ligand, these receptors transmit positive signals of differentiation, proliferation or migration, whereas in the absence of ligand, they turn into deadly weapons driving cells to commit suicide. Such a feature should lead cells expressing such receptors to become dependent on the presence of their ligand for survival.

A growing number of so-called dependence receptors are being identified: the low-affinity neurotrophin receptor P75^ntr^ (Rabizadeh *et al*, 1993), the androgen receptor AR (Ellerby *et al*, 1999), the DCC (for Deleted in Colorectal Cancer) receptors (Mehlen *et al*, 1998), RET (REarranged during Transfection) (Bordeaux *et al*, 2000), UNC5H (Llambi *et al*, 2001), Patched (Thibert *et al*, 2003), neogenin (Matsunaga *et al*, 2004) and integrins such as *α*_v_*β*_3_ and *α*_5_*β*_1_ (Stupack *et al*, 2001). Interestingly, whereas these receptors are known to be involved in the development of the nervous system when bound by their ligand, they each have the ability to trigger apoptosis in the absence of ligand. We propose to focus on the role of some of the receptors most extensively studied so far: the dependence receptors for netrin-1. Netrin-1 is a laminin-related molecule (Figure 1) initially discovered as a diffusible molecule produced by a ventral structure in the developing spinal cord, that is, floor plate, that attracts commissural axons (Serafini *et al*, 1994). Netrin-1 is a member of a family of homologous molecules like netrin-2, netrin-G1, netrin-G2 and netrin-4/*β*-netrin. Although next to nothing is known about these molecules, netrin-1 has been extensively described, particularly by Tessier-Lavigne and collaborators. As such, netrin-1 was shown to act as a chemoattractive or chemorepulsive cue for many migrating axons and neurons. This effect is believed to pass through two main families of type 1 transmembrane receptors: DCC and its homologue neogenin and the UNC5H – UNC5 homologue – receptors (UNC5H1, UNC5H2, UNC5H3, UNC5H4) (Figure 1). Because netrin-1 and its receptors have already been reviewed for their role in neuronal guidance (Tessier-Lavigne and Goodman, 1996; Mehlen and Mazelin, 2003; Mehlen and Fearon, 2004), we will mainly describe here the implications of netrin-1, DCC and UNC5H in apoptosis and consider the consequences on tumour escape mechanisms.

## ROLE OF DCC AND UNC5H AS DEPENDENCE RECEPTORS

The role of DCC and UNC5H as receptors for netrin-1, a molecule involved in axon guidance and neuron migration, suggested that signal transduction occurred only in the presence of the ligand netrin-1. Yet, we and others have shown that both DCC and UNC5H are much more complex. Indeed, we have suggested these molecules to act as dependence receptors, which are also active in the absence of their ligand. Various cell transfection or viral infection experiments have demonstrated that UNC5H or DCC, when expressed in the absence of netrin-1, can induce cell death, whereas the presence of netrin-1 is sufficient for blocking this proapoptotic activity (Mehlen *et al*, 1998; Forcet *et al*, 2001; Llambi *et al*, 2001; Liu *et al*, 2002; Tanikawa *et al*, 2003; Thiebault *et al*, 2003; Williams *et al*, 2003).

Little is known about the exact mechanism underlying the proapoptotic activity of DCC and UNC5H. However, both seem to be cleaved by major proteases of the apoptotic pathway, caspases. Caspases are cysteine proteases that cleave intracellular proteins on the carboxyl side of an aspartate residue. Caspases involved in apoptosis can be split into two groups: initiator caspases (e.g., caspase-8 and -9) and effector caspases (e.g., caspase-3, -6 and -7). Initiator caspases are activated by a death receptor pathway (caspase-8) or a mitochondrial pathway (caspase-9). Their activation initiates a proteolytic cascade that results in activation of the effector caspases, thus rapidly triggering the execution of apoptosis and ultimately leading to cell death.

**Figure 1:**
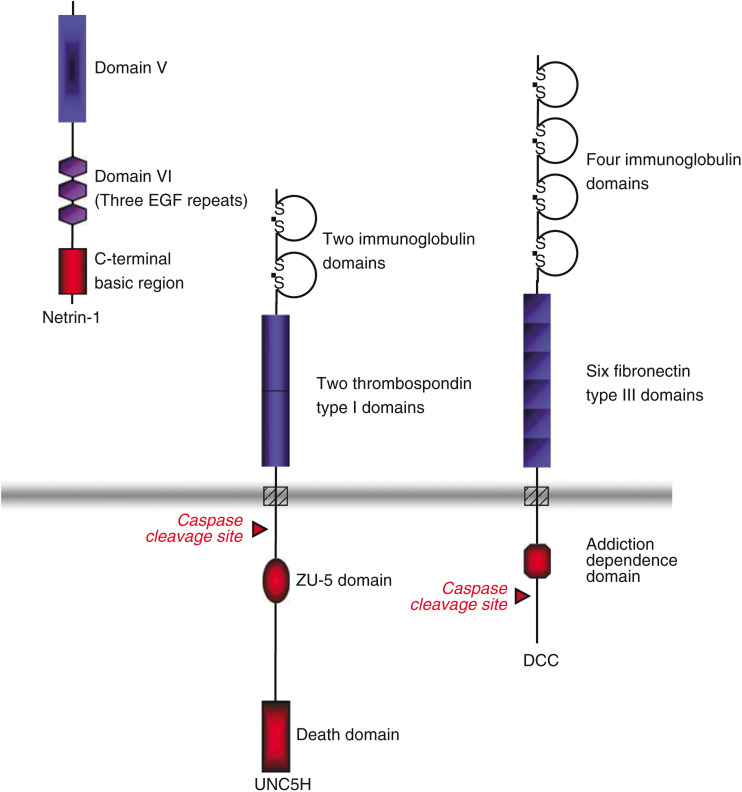
Structure of netrin-1 and netrin-1 dependence receptors. DCC is a type I transmembrane protein with an extracellular domain composed of four immunoglobulin domains, six fibronectin type III domains, a single transmembrane spanning region and a cytoplasmic domain including ADD. The four UNC5H receptors all have two immunoglobulin domains and two thrombospondin domains in the extracellular region and a ZU-5 domain (a domain of homology found in Zona Occludens-1 and UNC-5 protein). Their ligand, netrin-1, is a laminin-related secreted protein with V and VI domains (three epidermal growth factor domains) related to laminin and a positively charged carboxy (C)-terminal domain.

The cleavage sites of UNC5H and DCC receptors are recognised *in vitro* by caspase-3 (at position 412 for UNC5H and 1290 for DCC; see Figure 1), which does not necessarily imply that caspase-3 is responsible for the cleavage of these receptors *in vivo* (Mehlen *et al*, 1998; Llambi *et al*, 2001). Mutations of the cleavage site prevent the proapoptotic activity of these receptors, suggesting that cleavage is a prerequisite for cell death induction by releasing/exposing a proapoptotic domain named addiction dependence domain (ADD) lying in the intracellular domain of DCC or UNC5H.

In UNC5H, the ADD domain is thought to be located downstream of the caspase cleavage site and to encompass a death domain very similar to that of proteins like Fas and TNFR receptors, which are generally considered to mediate apoptotic signalling (Llambi *et al*, 2001). How the death domain of UNC5H induces apoptosis is yet unknown, even though it is probable that other death domain-containing proteins are required. Along this line, we recently demonstrated that UNC5H2 interacts with a death domain-containing serine/threonine kinase protein named death-associated protein kinase (DAPK) and that DAPK is required for UNC5H2-induced cell death (Llambi *et al*, 2005). Besides the death domain described above, UNC5H receptors harbour another domain, also involved in cell death, ZU-5. This domain is homologous to Zona Occludens-1, an intercellular junction protein involved in signalling. Recently, NRAGE, a protein of the MAGE (melanoma antigen) family known to be a regulator of apoptosis, has been identified as a specific binding partner of UNC5H1. The interaction between NRAGE and UNC5H1 could activate the apoptotic pathway by promoting the degradation of the survival protein XIAP (X-linked inhibitor of apoptosis), or by activating the c-Jun N-terminal kinase (JNK) pathway (Williams *et al*, 2003).

In DCC, the ADD is located upstream of the caspase cleavage site and is probably exposed through conformational changes after the caspase-dependent release of the C-terminal domain (Mehlen *et al*, 1998). The DCC ADD domain has been shown to interact with caspase-9 in the absence of netrin-1 (Mehlen *et al*, 1998; Forcet *et al*, 2001). As evidenced *in vitro* so far (Mehlen *et al*, 1998), this interaction is hypothesised to activate effector/downstream caspases, thus forming an amplification loop leading to more DCC cleavage and apoptotic cell death. Alternatively, other proteins may also interact with the ADD of DCC, as recently shown by Chen and colleagues. Indeed, DIP13 (DCC-interacting protein-13) has been shown to interact with DCC ADD and to mediate DCC-induced cell death (Liu *et al*, 2002).

Results obtained *in vivo* in netrin-1 knockout mice have confirmed that cells expressing DCC and UNC5H are dependent on netrin-1 for survival. In the absence of netrin-1, brainstem cells, especially those expressing DCC and/or UNC5H genes, undergo massive apoptosis (Llambi *et al*, 2001). Along the same line, while looking for a role of UNC5H in axon guidance in *Xenopus*, Tessier-Lavigne and co-workers (Hong *et al*, 1999) used a UNC5H mutant deleted of its death domain because expression of the wild-type receptor induced cell death.

The physiological relevance of the ‘negative’ face of these receptors in the absence of netrin-1 can be dual. Indeed, while DCC and UNC5H have been shown to impact positively on axon guidance by acting as receptors for netrin-1, a negative control by these receptors, that is cell death, can also occur. In this respect, the dynamic regulation of axons during CNS development may be a result of a combination of two opposite (negative and positive) control mechanisms. The negative effect would be a ‘surveillance’ mechanism capable of eliminating cells that are beyond the ligand source. In CNS cells, the growth and orientation of neurons would follow receptor-mediated chemoattraction/repulsion pathways (depending on the receptor expressed at the cell surface) in response to the source of netrin-1. This response is dependent on the capacity of receptors to bind netrin-1. When neurons migrate away from the netrin-1 source, receptors that have become ‘unoccupied’ may induce cell death, thus preventing cells to migrate to ‘unwanted’ sites.

**Figure 2:**
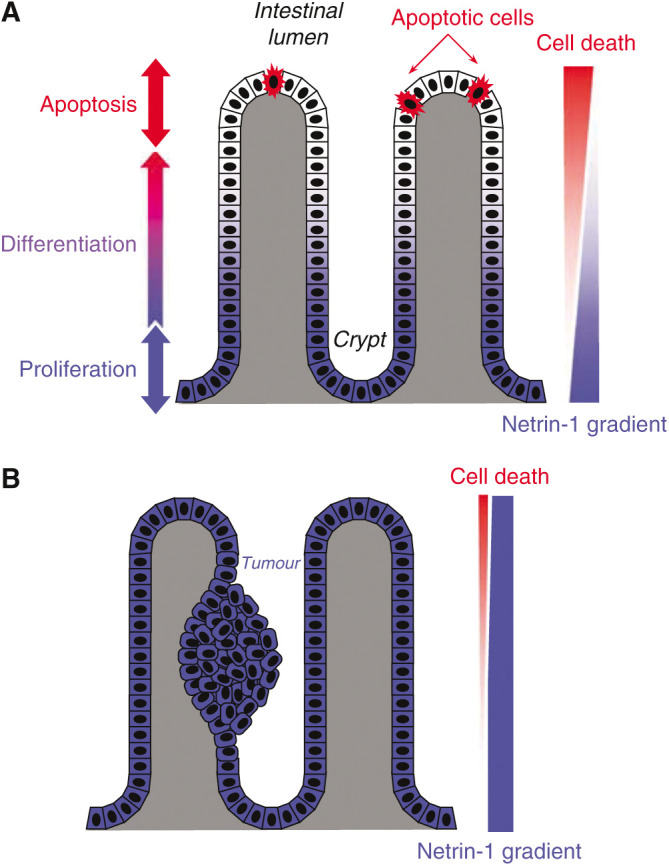
(**A**) View of normal intestinal villi. Cells are produced in the intestinal crypts at the base of the villi. As they differentiate, they migrate to the surface of the epithelium (intestinal lumen) where they are eventually eliminated. Netrin-1 is preferentially expressed at the base of intestinal crypts, whereas its receptor DCC is evenly distributed throughout the villi. Netrin-1 binding to DCC in crypt cells contributes to cell survival. On the contrary, the absence of netrin-1 at the villus surface induces apoptotic cell death. (**B**) View of intestinal villi overexpressing netrin-1. The even distribution of netrin-1 on the villus surface prevents apoptotic cell death, thus promoting tumour growth.

The second possible role *in vivo* is to regulate cellular lifespan, as recently proposed in intestinal villi. Indeed, the expression profiles of netrin-1 and DCC in the normal intestine and colon might suggest that cell survival regulation is dependent on the DCC/netrin-1 pair. In these tissues, the production of netrin-1 is restricted to the base of the intestinal crypt, whereas DCC is distributed throughout the villi (Hsu *et al*, 2001; Mazelin *et al*, 2004). This goes well with the classic view of highly proliferative cells in the base of the intestinal crypt that differentiate while moving to the distal part of the villi/crypt and that eventually detach and die or die and detach (Figure 2A). In this view, proliferating crypt cells that express DCC in a netrin-1-rich environment would be protected from cell death, whereas epithelial cells that have stopped proliferating to differentiate and move towards the villus tip would progressively be placed into a netrin-1-deprived environment, leading to apoptotic cell death (Figure 2A). In this respect, we have shown that overexpressing netrin-1 throughout the intestinal epithelium contributes to reducing apoptosis by approximately 50% (Mazelin *et al*, 2004). One may speculate that the apparent proximal-to-distal netrin-1 gradient may function as a regulatory system that limits the lifespan of cells that undergo (i) multiple proliferative steps within the crypt and (ii) repeated mechanical and chemical insults originating from the intestinal lumen, two conditions that may increase the risk of cell damage and resultant aberrant behaviour. Consequently, induction of cell death due to the absence of superficial netrin-1 expression, together with the mechanical detachment of cells in the lumen, may represent key factors for limiting the initiation of malignant transformation.

## ROLE OF DEPENDENCE RECEPTORS UNC5H AND DCC IN COLORECTAL TUMORIGENESIS

Due to the dependence mechanism described above, DCC and UNC5H could potentially be involved in tumour suppression. The receptor/netrin-1 pairs may indeed limit tumour development by inducing apoptosis of cells that have acquired transforming capacities. Any tumour cell submitted to an inadequate/abnormal environment (highly proliferative cells in an environment with limited and constant netrin-1 concentration, migration to other tissues because of metastatic propensities) would display unbound dependence receptors, thus triggering the proapoptotic activity of DCC and/or UNC5H, which would ultimately lead to cell death and subsequent tumour regression. In cancer, deletion of genes coding for DCC and UNC5H would induce loss of the proapoptotic signal, thus providing a selective advantage for tumour escape.

Along this line, in the early 1990s, the DCC gene was proposed as a putative tumour suppressor gene. Data supporting this proposal included observations that a DCC allele, located on chromosome 18q, was deleted in over 70% of colorectal cancers and a number of other tumours (Fearon *et al*, 1990; Cho *et al*, 1994). Interestingly, in most reports, allelic losses of 18q are infrequent in early-stage tumours (e.g., small adenomas), but are common in primary colorectal carcinomas and nearly 100% of hepatic metastases arising from colorectal primaries, implying that chromosome 18q loss of heterozygosity (LOH) may contribute more to progression rather than initiation of colorectal cancer. In more than 90% of primary colorectal cancers with LOH of chromosome 18q, DCC is included in the region of allelic loss (Fearon *et al*, 1990; Thiagalingam *et al*, 1996). Most studies have linked chromosome 18q LOH in colorectal cancers to a reduction in *DCC* expression both at the RNA level (Thiagalingam *et al*, 1996) and at the protein level (Goi *et al*, 1998). Loss of heterozygosity of chromosome 18q and/or decreased *DCC* expression have also been seen in various other cancers, including gastric, prostate, endometrial, ovarian, oesophageal, breast, testicular, glial, neuroblastoma and haematologic malignancies (see for review Mehlen and Fearon, 2004).

Chromosome 18q LOH has been associated with poor prognosis in colorectal cancer patients lacking lymph nodes or distant metastases at the time of surgery (so-called stage II), as well as in patients who have lymph nodes but no distant metastases at the time of surgery (stage III) (Jen *et al*, 1994; Ogunbiyi *et al*, 1998). In other studies, chromosome 18q LOH has also been associated with decreased responsiveness to 5-fluorouracil-based adjuvant chemotherapy regimens in stage III colorectal cancer patients (Watanabe *et al*, 2001; Barratt *et al*, 2002). Loss of DCC expression was often associated with poor prognosis and increased risk of metastasis (Shibata *et al*, 1996; Saito *et al*, 1999). The data indicating that 18q allelic loss and decreased *DCC* expression are associated with poor prognosis and possibly decreased response to adjuvant chemotherapy in colorectal cancer patients are interesting and of potential clinical significance. Nevertheless, the findings do little to establish whether *DCC* loss/inactivation is a critical factor in tumorigenesis or merely an epiphenomenon. Some evidence that *DCC* inactivation may in fact be associated with tumorigenic growth properties in colon and other cancers has been obtained. For example, introduction of an intact copy of chromosome 18 into a colorectal cancer cell line lacking endogenous *DCC* expression yielded detectable levels of *DCC* transcripts and resulted in suppression of growth in soft agar and tumorigenicity in nude mice (Tanaka *et al*, 1991). Also, ectopic expression of *DCC* in a tumorigenic keratinocyte cell line lacking endogenous *DCC* expression was shown to suppress tumorigenic growth of the cells in nude mice (Klingelhutz *et al*, 1993). Interestingly, in this study, it was observed that tumorigenic reversion was associated with loss of *DCC* expression and loss or rearrangement of the transfected *DCC* expression vector (Klingelhutz *et al*, 1993). Several more recent studies also indicate that restoration of *DCC* expression can suppress tumorigenic growth properties *in vitro* or in nude mice (Velcich *et al*, 1999; Kato *et al*, 2000). More recently, an interesting study suggested that the DCC/netrin-1 pair is important in endometrial carcinogenesis because (i) DCC is lost in nearly all the endometrial cancer cell lines tested and (ii) re-expression of DCC in these cell lines drives apoptosis, a phenomenon blocked by the presence of netrin-1 (Kato *et al*, 2004). However, beside these numerous arguments towards a role of DCC as a tumour suppressor gene, various concerns, such as the rarity of point mutations identified in *DCC* coding sequences, the absence of *DCC* germline mutations involved in a heritable cancer predisposition or the lack of a tumour predisposition phenotype in mice heterozygous for *Dcc* inactivating mutations, together with the presence of other known and candidate tumour suppressor genes on chromosome 18q have raised questions about the role of DCC. However, as discussed in detail in another review (see Mehlen and Fearon, 2004), none of these concerns appears sufficient to discard the hypothesis that DCC acts as a tumour suppressor gene.

Similarly, it has been observed that the expression of UNC5H is strongly reduced in more than 90% of colorectal cancers, as well as in many other tumours. In colorectal cancers, this reduction has been mostly associated with UNC5H1 and UNC5H3 (Thiebault *et al*, 2003). In this case, allelic losses have been observed, but it is tempting to attribute the loss of UNC5H expression to epigenetic mechanisms such as promoter methylation (Thiebault *et al*, 2003). Moreover, UNC5H2 has been shown to be a direct transcriptional target of the p53 tumour suppressor gene whose proapoptotic activity is known to be dependent on the p53-dependent expression of UNC5H2 and can be antagonised by the presence of netrin-1 (Tanikawa *et al*, 2003). Moreover, several *in vitro* experiments have shown that DCC and UNC5H compromise the hallmark features of cell transformation: anchorage-independent growth and ability to invade through a Matrigel matrix (Thiebault *et al*, 2003; Mehlen and Fearon, 2004).

To evaluate the role of DCC and UNC5H receptors in tumorigenesis and to avoid the usual bias of using inactivating strategies (knockout) in which both the positive (netrin-1-dependent signals) and the negative (apoptosis in the absence of netrin-1) pathways are inactivated, we have developed an alternative method. We have forced mice to overexpress netrin-1 in the intestinal epithelium, a model that has enabled us to prevent receptor-induced cell death (Figure 2B). Indeed, it has been reported that the targeted overexpression of netrin-1 throughout the digestive tract can produce approximately 50% cell death inhibition in the intestinal epithelium (Mazelin *et al*, 2004). This inhibition of cell death, in agreement with the model proposed for cell lifespan regulated by netrin-1 control of DCC-induced cell death, is associated with the formation of numerous focal hyperplasias (compared to control mice) and of adenomas (Mazelin *et al*, 2004). Thus, inhibition of cell death by netrin-1 is associated with an increased initiation of colorectal tumorigenesis. Because DCC loss in humans is very often considered as a late event, netrin-1-overexpressing mice are backcrossed in a mouse model in which colorectal tumorigenesis is initiated by a mutation in adenomatous polyposis coli (APC) (Fodde *et al*, 1994). Interestingly, while APC mutated mice develop low-grade adenomas, the development of tumours in the APC/netrin-1 mice was pushed towards high-grade adenoma and adenocarcinoma (Mazelin *et al*, 2004). The above results demonstrate that blocking cell death induced by netrin-1 overexpression is associated with both an initiation and a progression of colorectal tumorigenesis (Mazelin *et al*, 2004), thus confirming that dependence receptors expressed in the digestive tract play a role in tumorigenesis.

Several key issues remain to be answered. First, which is the receptor involved? Indeed, whether it is DCC or UNC5H1-3 remains to be shown. Interestingly, UNC5H and DCC proteins display a distinct localisation inside the intestinal villi: UNC5H2 and DCC appear to be present along the whole intestinal villi, while UNC5H3 is present only within the crypt (Mazelin *et al*, 2004). Together with the fact that overexpression of netrin-1 in the mouse model described above shows two ‘time window’ effects – one during early phase of tumorigenesis, as illustrated by the adenomas and focal hyperplasias observed in these mice, and one late effect with the formation of adenoma-carcinomas in the APC/netrin-1 mice – this would point to regulatory functions of these dependence receptors at different times of colorectal tumorigenesis. A second question is related to netrin-1 expression in human colorectal tumours, as it would be expected that a similar selective advantage for a tumour is either to lose receptor expression (as it occurs for DCC and/or UNC5H) or to gain netrin-1 expression. A preliminary set of data has shown that overexpression of netrin-1 is only rarely associated with human colorectal cancer –7% of the tested tumours (Mazelin *et al*, 2004) – therefore suggesting that gain of netrin-1 or loss of the receptor does not represent a similar selective advantage. Further work will have to analyse whether this effect is restricted to colorectal cancers or if it is general to cancer development.

More work has to be performed to prove that the effect seen in netrin-1-overexpressing mice is indeed due to the inhibition of DCC- or UNC5H-induced cell death by netrin-1 and not to the fact that netrin-1 overexpression leads to overstimulation of alternative receptors, such as integrins or A2b, nor to the fact that netrin-1 has a positive effect on tumour development due to chemoattrative/chemorepulsive activities. Indeed, it was recently suggested that UNC5H2 plays a role in embryonic angiogenesis, even though this probably occurs independently of netrin-1 (Lu *et al*, 2004). It was also shown that netrin-1 can enhance angiogenesis in a DCC/UNC5H-independent mechanism (Park *et al*, 2004). However, it is fair to say that netrin-1 receptors such as DCC and UNC5H are probably involved in tumour growth control and can thus be seen as tumour suppressors, even though their proactivities in low netrin-1 presence largely differ from the growth inhibitory activity of other known tumour suppressors such as Rb that constitutively repress cell cycle. DCC and UCN5H only acquire their suppressive activity when cells grow abnormally and/or migrate to ‘unwanted’ sites where the ligand concentration is low. This is why we have named these receptors ‘conditional’ tumour suppressors. Whether these conditional tumour suppressors or their proapoptotic signalling pathways will turn out to be interesting targets for cancer therapy remains to be investigated.
